# Editorial: Chronic airway diseases, lung cancer, and their interaction

**DOI:** 10.3389/fmed.2023.1201894

**Published:** 2023-05-31

**Authors:** Yi Liu, Youming Zhang

**Affiliations:** ^1^Department of Pulmonary and Critical Care Medicine, Shandong Provincial Hospital Affiliated to Shandong First Medical University, Jinan, Shandong, China; ^2^Section of Genomic and Environmental Medicine, National Heart and Lung Institute, Imperial College London, London, United Kingdom

**Keywords:** asthma, COPD, infection, lung cancer, microbiome

Chronic respiratory diseases include chronic airway diseases, chronic cough, pulmonary fibrosis, pulmonary infection, and lung cancer. Asthma and chronic obstructive pulmonary disease (COPD) are the most common chronic respiratory diseases, both are inflammatory diseases in small airways. Asthma affects 300 million people in the world (1). Important components of the asthma syndrome include severity, age of onset and the presence of eosinophilia and IgE-mediated allergy to inhaled proteins. Type 2 immune responses are dominant in asthma in Westernized societies, but most cases of asthma in the developing world are non-atopic (2). COPD is also a heterogeneous disease and a leading cause of death and disability worldwide. It characterizes as persistent airflow obstruction and respiratory symptoms. The disease is caused by exposure to inhale cigarette smoke and air pollutants, in combination with genetic, developmental, and social factors (3). Lung cancer can be classified into two broad histologic classes: small cell lung carcinomas (SCLC) and non-small cell lung carcinomas (NSCLC). It is the leading cause of cancer mortality worldwide. Smoking is also the major cause of lung cancer (about 90% of cases). Radon gas, asbestos, air pollution exposures, and chronic infections can also contribute to lung carcinogenesis (4).

With the advanced omics technologies, clinical managements for chronic airway diseases and lung cancer have been revolutionized. For asthma, IgE-specific antibodies, leukotriene receptor antagonists, Th2 type cytokines, therapies against tumor necrosis factor (TNF), vitamin D, probiotics, pathogen-associated molecular patterns (PAMPs) and toll-like receptors (TLRs) agonists, and interferons (IFNs) are all possible treatment means (5). Inhaled corticosteroids (ICS) are still the mainstay for asthma treatment and short-acting beta-agonists (SABAs) can also rapidly reduce airway bronchoconstriction. Although these advanced biologic therapies have reduced the exacerbations rates of moderate to severe asthma by 50% (6), two-thirds of patients with severe asthma treated with biologics continue to have uncontrolled diseases (7). For COPD, there's currently no cure for the disease, but treatments can help to slow the progression of the condition. The effective managements of COPD include stopping tobacco, inhalers, pulmonary rehabilitation, surgery, or lung transplant. For lung cancer, treatments include surgery, radiation therapy, chemotherapy, targeted therapy, and immunotherapy (4). Treatments with tyrosine kinase inhibitors (TKIs) and immune checkpoint inhibitors (ICPIs) have been emerged as valuable therapies for some lung cancer patients. Combining with other traditional therapeutic options, targeted therapies and immunotherapies show great potential in lung cancer treatments.

Personized medicine or precision medicine requires to understand host genetic factors and environmental factors. Chronic airway diseases and lung cancer are caused by combinations of the two factors. Asthma and COPD exacerbations are often linked to the bacterial and viral infections. Human rhinovirus (HRV) and non-typeable Haemophilus Influenzae (NTHi) infections contribute to airway diseases (8, 9). Lower respiratory tract infections (LRTI) are leading causes of morbidity and mortality in the world, accounting for 4.4% of deaths among all ages (10). *Klebsiella pneumoniae* (Kp) is a nosocomial gram-negative bacterial pathogen and can cause community-acquired bacteraemia, liver abscess, urinary tract infection and pneumonia. In this special collection Yang et al. identified 49 patients had sequence type 11 (ST11) from 139 patients infected *Klebsiella pneumoniae*. Patients with ST11 Kp infection can increase mortality in hospital and the infection could be regarded as an independent risk factor for mortality in respiratory ward. The human microbiota plays an important role in immune system development and tissue homeostasis. Microbiome in human lungs exhibits a low biomass and is dominated by dynamic fluxes of microbial clearance and immigration. Respiratory diseases can disrupt the microbial-host interface and affect disease development (11). Zhu et al. presented a cross-sectional study of sputum samples from 36 healthy volunteers and 34 patients with an acute exacerbation of chronic obstructive pulmonary disease (AECOPD) in Inner Mongolia area in China with high-throughput sequencing of 16S rDNA. The airway microbiota of the AECOPD population was different from that of the healthy population. The diversity of airway microbiota was lower than that of the healthy population. Long-term use of ICS plus long-acting beta agnoist (LABA) leads to lower alpha diversity in AECOPD patients. In a meta-analysis, Liu et al. analyzed testosterone deficiency in COPD patients. They found uncertainty for improving the quality of life of COPD patients with anabolic-androgenic steroids (AASs) treatment, suggesting longer and larger future studies for better clarifying the efficacy of AASs. Dendritic cells (DCs) are important bridges to connect innate and adaptive immunity. Xuan et al. presented a systematic review on the roles in respiratory diseases such as lung carcinoma, asthma, pulmonary fibrosis, and pulmonary Langerhans cell histiocytosis, COPD and microbial infection. Hui et al. presented another meta-analysis to summarize the association between polymorphism of angiotensin-converting enzyme (*ACE*) gene and asthma risk. The genotypes DD and ID of *ACE* gene associated with circulating ACE levels. High circulating ACE levels contributed to the development of asthma. Acupuncture at the sphenopalatine ganglion (SPG) for seasonal allergic rhinitis (SAR) is widely applied to treat seasonal allergic rhinitis in China, but the effectiveness is not clear. Wang et al. provided a protocol for a parallel-design, three-armed, patient-assessor blinded randomized controlled trial. The results of the clinical trials would bring the outcomes of the effectiveness of acupuncture treatment on SAR.

Lung cancer is still the most common cancer in China. Li et al. investigated the epidemiology of lung cancer in China from 1990 to 2019. They identified top five risk factors including smoking, ambient particulate matter pollution, second hand smoke, high fasting plasma glucose, and household air pollution from solid fuels. The results provided valuable clues for the prevention and treatment of lung cancer in China. Liang et al. found rapid on-site evaluation (ROSE) examination can increase the diagnostic accuracy of malignant diseases during endobronchial ultrasound-guided transbronchial needle aspiration (EBUS-TBNA). The method can reduce the number of intraoperative punctures. Shen et al. analyzed patients with multiple lung nodules, they applied four factors including chronic inflammation history, human Th cell, imaging vascular convergence sign and mixed ground-glass nodules (GGNs) to assess GGNs. They established a prediction model that can greatly improve the accuracy of malignancy or benignancy prediction of sub-centimeter pulmonary GGNs. Immune checkpoint inhibitors (ICIs) have brought revolutionary breakthroughs to lung cancer, but the therapy has adverse events such as hepatitis, nephritis, dermatitis, and myocarditis. Zhou et al. reported one case of myocarditis with the treatment of PD-L1 inhibitor. The myocardial injury in this case happened in a short time and returned to normal after applying glucocorticoids therapy.

This Research Topic contains 10 valuable articles for the chronic airway diseases and lung cancers and the interaction. Further research in the field can provide more exciting evidence for understanding the pathophysiology of the diseases. Novel therapeutic means will give respiratory patients better, and personalized care in future.

## Author contributions

YL and YZ drafted the manuscript, revised the version, and gave approval for the submission. All authors contributed to the article and approved the submitted version.
